# Economic burden of diabetes in Brazil in 2014

**DOI:** 10.1186/s13098-019-0448-4

**Published:** 2019-07-02

**Authors:** Luciana Ribeiro Bahia, Michelle Quarti Machado da Rosa, Denizar Vianna Araujo, Marcelo Goulart Correia, Roger dos Santos dos Rosa, Bruce Bartholow Duncan, Cristiana Maria Toscano

**Affiliations:** 1 Internal Medicine Department, Institute for Health Technology Assessment, State University of Rio de Janeiro, Boulevard 28 de Setembro, 77-Vila Isabel, Rio de Janeiro, RJ 20551-030 Brazil; 2 Biostatistics and Bioinformatics Department, National Institute of Cardiology, Rio de Janeiro, Brazil; 3 Social Medicine Department, Federal University of Rio Grande do Sul, Porto Alegre, Brazil; 4 Graduate Studies Program in Epidemiology, Federal University of Rio Grande do Sul, Porto Alegre, Brazil; 5 Collective Health Department, Institute for Health Technology Assessment, Federal University of Goiânia, Goiânia, Brazil

**Keywords:** Type 2 diabetes, Cost analysis, Cost studies, Public health

## Abstract

**Background:**

Diabetes and its complications produce significant clinical, economic and social impact. The knowledge of the costs of diabetes generates subsidies to maintain the financial sustainability of public health and social security systems, guiding research and health care priorities.

**Aims:**

The aim of this study was to estimate the economic burden of diabetes in Brazilian adults in 2014, considering the perspectives of the public health care system and the society.

**Methods:**

A prevalence-based approach was used to estimate the annual health resource utilization and costs attributable to diabetes and related conditions. The healthcare system perspective considered direct medical costs related to outpatient and hospitalization costs. The societal perspective considered non-medical (transportation and dietary products) and indirect costs (productivity loss, disability, and premature retirement). Outpatient costs included medicines, health professional visits, exams, home glucose monitoring, ophthalmic procedures, and costs related to end stage renal disease. The costs of hospitalization attributed to diabetes related conditions were estimated using attributable risk methodology. Costs were estimated in Brazilian currency, and then converted to international dollars (2014).

**Results:**

Based on a national self-reported prevalence of 6.2%, the total cost of diabetes in 2014 was Int$ 15.67 billion, including Int$ 6.89 billion in direct medical costs (44%), Int$ 3.69 billion in non-medical costs (23.6%) and Int$ 5.07 billion in indirect costs (32.4%). Outpatient costs summed Int$ 6.62 billion and the costs of 314,334 hospitalizations attributed to diabetes and related conditions was Int$ 264.9 million. Most hospitalizations were due to cardiovascular diseases (47.9%), followed by diabetes itself (18%), and renal diseases (13.6%). Diet and transportation costs were estimated at Int$ 3.2 billion and Int$ 462.3 million, respectively.

**Conclusions:**

Our results showed a substantial economic burden of diabetes in Brazil, and most likely are underrated as they are based on an underestimated prevalence of diabetes. Healthcare policies aiming at diabetes prevention and control are urgently sought.

**Electronic supplementary material:**

The online version of this article (10.1186/s13098-019-0448-4) contains supplementary material, which is available to authorized users.

## Background

Noncommunicable diseases (NCDs) are a major challenge for sustainable development and represent about 80% of the mortality in Brazil [1]. Diabetes mellitus (DM) is a considerable cause of morbidity, mortality and health-care costs in the world [2]. It imposes substantial costs on patients, their families, health systems, and national economies because of direct costs of treatment and loss of work and wages [3]. Increased prevalence and longer life expectancy are the driving forces behind the growing economic burden of diabetes.

The International Federation of Diabetes (IDF) estimated that in 2017 there were 424.9 million people with diabetes aged 20–79, and 4 million deaths attributable to diabetes, resulting in global health expenditure of USD 727 billion. The number of individuals with diabetes is expected to rise by 48% by 2045. Of these, 79% were estimated to be living in low- and middle-income countries [4]. Brazil, a large high-middle income country, ranks among the top five countries with the largest number of individuals with diabetes and evidence suggests that diabetes prevalence has increased by 61.8% in the last 10 years [5].

Better understanding the economic burden of diabetes and related complications is of paramount importance not only to mobilize society and inform policy makers, but also to help determine the cost-effectiveness of interventions for disease prevention and control. The aim of this study was to estimate the economic burden of diabetes and related diseases in adults in 2014, considering the perspectives of the Brazilian public health system and society.

## Research design and methods

### Study perspective, costing methods and cost components

The Brazilian public healthcare system (SUS) perspective included direct medical costs related to outpatient management, procedures, and hospitalization. The societal perspective considered in addition, non-medical (transportation and dietary products) and indirect costs (productivity loss, disability, and premature retirement).

Depending on the cost component, a combination of micro-costing and gross-costing was considered, as further detailed below.

Several data sources were considered, including the 2013 National Health Survey (NHS) [6], the National Public Hospitalization Information System(SIH/SUS) [7], the National Outpatient Information system (SIA/SUS) [8], the National price listings including the standardized tables of costs of consultations, exams and procedures (SIGTAP) [9], the cost of public purchases for medicines [10] and the National Household Survey for average wage income [11]. Data from a national cost of illness study on outpatient health resource utilization and productivity loss was also used [12].

All costs were estimated in Brazilian Reais and then converted into International dollars (Int$) considering the 2014 purchasing power parity (PPP) (1 Int$ = 1.748 BRL) [13]. Overall costs and per patient costs are presented, by cost component and type.

### Prevalence of diabetes

A prevalence-based approach was used to estimate the annual health resources utilization and costs attributable to diabetes and related diseases. The National prevalence of self-reported diabetes was obtained from a National Health Survey representative of the Brazilian population [6], which estimated the diabetes’ prevalence to be 6.2% in the adult population aged 18 years or older. Prevalence estimates were obtained for each one of all the 27 states (Additional file 1: Table S1). In order to estimate the number of individuals with diagnosed diabetes in 2014, diabetes prevalence estimates were combined with the national population estimates, by age group, gender and state [14]. The total adult population (20 years and older) in 2014 was estimated at 137,640,060 inhabitants.

## Direct medical costs

### Hospitalizations

Raw hospitalization data were obtained from the files of the Brazilian Hospital Information System (SIH/SUS) [7]. This database is the national control system of payment of services provided by all public and private hospitals to SUS.

Hospitalizations were categorized into two groups: DM itself and chronic complications of DM and other related diseases. The first group corresponds to hospitalizations with main diagnosis ICD-10 E10 to E14. The second group includes microvascular (retinopathy, nephropathy, and neuropathy) and macrovascular (coronary heart disease, cerebrovascular disease, and peripheral arterial disease) complications of DM, and selected infectious and neoplastic diseases for which diabetes is a known risk factor. In this second group the medical conditions were represented by 66 diagnoses reported as being the main cause of hospitalization, as coded by the International Classification of Diseases, 10th revision (ICD-10) (Additional file 1: Table S2).

We used an attributable risk methodology, which is based on population etiological fractions [15]. The relative risk or odds ratio of hospitalization of individuals with diabetes when compared to individuals without diabetes was combined with the estimated proportion of the population with diabetes to calculate the etiological fraction for each condition considered. The etiologic fraction for each medical condition represents the proportion of hospitalizations due to this condition that is attributable to diabetes, considering the following formula:

$${\text{PARi}} = \left[ {{\text{P}} \times \left( {{\text{RRi}} - 1} \right)} \right]/\left[ {{\text{P}} \times \left( {{\text{RRi}} - 1} \right) + 1} \right]$$

where PAR i is the population attributable risk fraction for the medical condition “i” due to diabetes; P represents the prevalence of diabetes; and RRi is the relative risk or the odds ratio of the medical condition “i” for people with diabetes compared to those without the disease.

As individuals with diabetes who are unaware of the disease may also be hospitalized due to diabetes or its complications, we considered the prevalence of undiagnosed diabetes for hospitalization costs. To account for undiagnosed diabetes, the prevalence of self-reported diabetes was multiplied by a factor of two, based on recent evidence from the Brazilian literature indicating that half of the individuals with diabetes diagnosis by laboratory confirmation were unaware of their disease [16, 17].

The relative risks for all conditions considered were obtained from the literature (Supplemental Table S2), representing the health service-related risk estimates associated with the hospitalization of individuals with diabetes. Total hospitalization costs attributed to diabetes and average cost per hospitalization by diagnostic groups (i.e., diabetes mellitus, cardiovascular disease, renal disease, eye disease, neurological diseases, infectious diseases and neoplasms) were estimated.

### Outpatients costs

Outpatient costs included patient management costs, and costs related to ophthalmic procedures and the management of end stage renal disease (ESRD). It included utilization of the following healthcare resources: medicines, exams, health professional visits, insulin syringes and home glucose monitoring (lancets and test strips). Resource utilization was obtained from a previous national cost-of-illness study [12]. The health resources were then multiplied by unit costs to obtain costs of outpatient management.

Unit costs of medicines were estimated based on the price of medicines included in the government diabetes treatment package (Farmácia Popular) [18], the average price of other medicines purchased by the government in 2014 [10], and regulated market prices for out-of-pocket medicines (manufacturer price plus tax) [19]. The costs of health professional visits, exams and procedures were obtained from official reimbursement SUS’s tables [9].

As outpatients with ESRD due to diabetic nephropathy and retinopathy incur with additional high costs procedures and medications, these costs were estimated separately. For patients with ESRD we considered the costs of dialysis and specific medicines. Resource utilization and costs of dialytic procedures in 2014 were obtained from the government outpatient information system (SIA/SUS) [8]. The prevalence of diabetes among all ESDR patients undergoing dialysis was considered as 29%, as reported by a national study conducted by the Brazilian Society of Nephrology [20]. The use of medicines and supplements (erythropoietin, iron, calcitriol, cinacalcet, paricalcitol and sevelamer) were obtained from a national study based on official government records [21]. Recommended doses and quantities were those proposed by National Clinical Protocols and Therapeutic Guidelines of the Ministry of Health [22–24].

For patients with diabetic retinopathy we considered all ophthalmic procedures related to diagnosis and treatment (fundoscopic exams, fluorescein angiographies, laser photocoagulation, intravitreal injections, cataract extractions). In addition, we also considered some selected inpatient procedures (cataract extractions and vitrectomies). A panel of specialists in retinal and vitreous diseases from public reference centers was established and consulted to identify the prevalence of individuals with diabetes performing such procedures. Considering these estimates, we were able to estimate the number of procedures performed in one year, which was then multiplied by their respective unit costs [9].

### Direct non-medical costs

Non-medical costs included patients’ expenses with dietary products and transportation for consultations and exams. Both were estimated from the primary study on diabetes cost [12].

The annual expenses with dietary products were estimated considering the expenditure reported in the previous month, which was then multiplied by 12. This value was then adjusted for 2014 considering annual inflation rates [25].

For transportation, we assumed that each individual would incur in average in one round-trip local travel to the healthcare service for each and every reported health professional consultation or laboratory tests performed. Due to the wide variety of urban means of transportation used (bus, train, subway, car), a local round trip cost was assumed as that of two regular bus fares considering the national average bus fare in all states in 2014 (Int$ 3.37) [26].

### Indirect costs

Productivity loss of patients and their companions was estimated through the reported absenteeism (hours/days) and premature retirement (years) due to diabetes and associated disability. Human capital approach for productivity loss estimates was based on the National average wage income in 2014 (R$1052/Int$ 601.9) [11], which served as a basis for calculating the value of the working hour. In accordance with the Brazilian labor legislation, the retirement age for the general population is 65 years for men and 60 years for women [27]. For early retirement estimates, we considered the adult population with diabetes, aged between 30 and 70 years old. Productivity loss associated with early retirement was calculated applying the prevalence of self-reported retirement (7.4%) due to diabetes and its complications from Bahia et al. [12].

## Results

Considering an estimated 9.2 million adult individuals with diabetes in Brazil, and assuming that all of them would be on treatment, the total estimated cost of diabetes in Brazil in 2014 in the societal perspective was Int$ 15.67 billion, of which Int$ 6.89 billion were direct medical costs (44%), Int$ 3.69 billion were direct non-medical costs (23.6%), and Int$ 5.07 billion were in indirect costs (32.4%).

When considering the perspective of the public healthcare system, diabetes costs summed Int$ 6.89 billion, of which Int$ 6.1 billion were outpatient management costs, Int$ 264.9 million were due to hospitalizations, and Int$ 476.8 million and Int$ 47.3 million of procedures related to ESRD and diabetic retinopathy, respectively.

### Costs of hospitalizations attributed to diabetes

There were 131,372 hospitalizations due to diabetes and 182,962 due to diabetes related diseases in 2014. Total hospitalization costs summed Int$ 264,912,885, most of which due to cardiovascular diseases (47.9%), followed by diabetes itself (18%), and renal diseases (13.6%). The average cost per hospitalization was higher for cardiovascular diseases (Int$ 1529) and renal diseases (Int$ 1601) (Table 1).

**Table 1:** Hospitalizations costs due to diabetes and related conditions in the Public Healthcare System (SUS), Brazil/2014

Diabetes and related diseases	Costs per person (Int$)^a^			Total costs—Brazil (Int$)		
	Men	Women	All	Men	Women	All
Diabetes mellitus	383	349	364	22,751,869	25,116,678	47,868,548
Attributed to diabetes						
Cardiovascular diseases^b^	1760	1300	1529	72,679,758	54,189,832	126,869,590
Renal diseases	1696	1488	1602	20,883,927	15,253,469	36,137,396
Eye diseases	700	567	621	5,927,851	7,002,473	12,933,324
Neurological diseases^c^	696	657	679	10,337,988	7,794,401	18,132,390
Infectious diseases^d^	642	610	624	6,007,232	6,716,031	12,723,263
Neoplasms^e^	1194	1214	1207	3,302,995	6,945,379	10,248,374
Total	956	746	845	141,891,620	123,018,263	264,912,885

*SUS* National Unified Health System (Sistema Único de Saúde)

^a^1 Int$ = 1.74 Brazilian Reais

^b^Coronary heart disease and cerebrovascular disease

^c^Diagnoses related to diabetic neuropathy

^d^Urinary and respiratory infections

^e^Breast, endometrial, pancreas, colorectal, hepatocarcinoma, cholangiocarcinoma

### Outpatient costs

Total outpatient costs were Int$ 9.8 billion, including Int$ 6.1 billion (62.2%) in medical costs and Int$ 3.7 billion (37.7%) in non-medical costs (Table 2). The largest share of outpatient direct costs was for medicines (45%), followed by dietary products (33%).

**Table 2:** Direct outpatient costs due to diabetes, Brazil/2014

Cost components	Costs per person/year (Int$)^a^	Total costs (Int$)	% of direct costs
Medicines	480.8	4,448,916,269	45.39
Insulin syringes	51.5	194,414,077	1.98
Health professional visits	76.2	705,386,372	7.19
Lab tests	49.2	455,750,648	4.65
Home glucose monitoring (lancets and test strips)	93.7	297,332,316	3.03
Total medical costs	751.4	6,101,799,682	62.26
Diet	349.7	3,236,104,918	33.03
Transportation	100.7	462,346,326	4.71
Total non-medical costs	450.4	3,698,451,244	37.74
Total direct costs (medical and non-medical)	1201.9	9,800,250,926	

^a^1 Int$ = 1.74 Brazilian reais

The annual cost per patient would be Int$ 1201.9, being Int$ 751.4 of direct medical costs and Int$ 450.4 of non-medical costs.

In Brazil, the number of individuals undergoing dialysis (hemodialysis and peritoneal dialysis) in 2014 was approximately 112,004, of which 29% (n = 32,481) had diabetes diagnosis [18]. The annual costs of dialysis procedures in the population with diabetes summed Int$ 476.8 million and the annual cost per patient was estimated as Int$ 13,005. The proportion of patients under dialysis using high costs medicines and supplements is presented in Table 3. For these medicines the estimated annual costs per patient summed $1003, totaling $14,038 related to ESRD (dialysis plus medicines).

**Table 3:** Direct outpatient costs related to end stage renal disease in patients with diabetes, Brazil/2014

Cost components	Costs per person/year (Int$)^a^	Total costs (Int$)
Dialysis (n = 32,481)		
Dialysis sessions, preparation and maintenance of arteriovenous fistulas, catheter implant and removal, maintenance and accompaniment of peritoneal dialysis	13,005	422,433,440
Erythropoietin (77%)^b^	343.8	8,598,298
Parenteral iron (53%)	101.5	1,747,310
Sevelamer (40%)	1662	21,602,337
Calcitriol (27%)	2595	8,697,590
Cinacalcet (4%)	7342	9,539,679
Paricalcitol (3%)	4313	4,203,290
Medicines (weighted average)	1033	54,366,108
Total costs	14,038	476,799,548

^a^1 Int$ = 1.74 Brazilian reais

^b^Percentage of use among patients on dialysis [20]

We estimated that 3,591,728 fundoscopic exams, 138,769 fluorescein angiographies, and 77,471 laser photocoagulation procedures were performed in adults with diabetes in public health units in 2014. Similarly, out of the surgical procedures, we estimated that 17,604 cataract extractions and 4318 vitrectomies were conducted in adult individuals with diabetes. Intravitreal injections were performed in 5863 individuals with diabetes (Table 4). The largest cost was that of the 3.5 million fundoscopic exams carried out in one year and the most costly procedure was vitrectomy.

**Table 4:** Direct outpatient costs related to ophthalmic procedures in patients with diabetes, Brazil/2014

Cost components	Number of procedures in patients with diabetes	Total costs (Int$)^a^
Fundoscopic exams	3,591,730	30,234,329
Retinal angiographies	138,769	2,897,743
Laser photocoagulation	77,471	2,909,988
Intravitreal injections^b^	5863	278,114
Cataract extractions	17,604	5,049,953
Vitrectomies	4320	5,946,690
Total costs		47,316,819

^a^1 Int$ = 1.74 Brazilian reais

^b^No mention of the medicines used for intravitreal injections

### Indirect costs

Productivity loss due to absenteeism and early retirement added Int$ 5.07 billion/year. The majority of this burden (64%) comes from the early retirement of individuals with diabetes who leave the workforce because of diabetes-associated disability. Considering average annual earnings of Int$ 7.223,13 per patient, we estimated a total of Int$ 3.2 billion of foregone earnings due to disability in 2014. This was reported by 7.4% of adults with diabetes, in average at the age of 53 years [12].

The total costs related to absenteeism was Int$ 1.8 billion. There was 89% of patients reporting loss of work days and 29.9% reporting a companion that lost work days, resulting in an estimated annual cost of Int$ 188.3 and Int$ 100 per patient and companion, respectively.

## Discussion

In Brazil the economic burden of diabetes for the society was Int$ 15.67 billion in 2014, of which 44% were direct medical costs, 23.6% were direct non-medical costs, and 32.4% were due to productivity loss. Economic costs of diabetes thus represented 0.52% of Brazilian gross domestic product (GDP) in 2014 [28]. From the perspective of the public healthcare system (i.e., not considering indirect costs and non-medical direct costs), the economic cost of diabetes was Int$ 6.9 billion. Our results demonstrate that public expenditures with diabetes and its complications alone represents 5.9% of the total public health expenditures (Int$ 116.73 billion) in Brazil in 2014 [29].

Bommer et al. estimated the global economic burden of diabetes in 2015 at US$1.31 trillion or 1.8% of world GDP, being on average, larger in low and middle-income countries, when compared to high-income countries [30]. The IDF Atlas estimated that approximately 11% of global health expenditure was dedicated to diabetes, and the average health expenditure per person with diabetes worldwide in 2014 ranged from Int$1583 to Int$ 3110, considering overall public and private expenditures [31]. This average was estimated to be lower (Int$ 1382) in South and Central American countries. In our study, just the annual direct costs per person (medical and non-medical) in the public health system summed Int$ 1201, without taking into account indirect and hospital costs.

Our estimates are based on a self-reported diabetes prevalence of 6.2%. Prevalence rates increased with age, varying from 0.6%, for those aged 20 to 29, to 19.9% for people aged 65–74. This prevalence rate contrasts with the recent results of the Brazilian Longitudinal Study of Adult Health (ELSA-Brazil), a cohort study of 15,105 civil servants aged 35–74, that reported overall prevalence of diabetes diagnosed by laboratory tests of 19.7% in adults, reaching 35% for those aged 65–74 [17]. We have evidence to believe that, considering the various barriers to access public health services and based on national studies, diabetes is still an under diagnosed condition in Brazil [17, 32], and thus the calculated diabetes burden is likely to be underestimated. This is further corroborated by recent evidence from the International Diabetes Federation, which reported globally an estimated 50% of adults with diabetes undiagnosed in 2017 [4].

Most of direct medical costs expenditures was for the purchase of medicines. In Brazil, a governmental pharmaceutical program (Farmácia Popular) was implemented in 2011 to increase access to medications for treatment of chronic diseases, including diabetes and hypertension [18]. Through this program a standardized package of medicines is distributed free of charge through thousands of private pharmacies throughout the country [33]. Nonetheless, it has been estimated that as much as 25% of individuals with diabetes purchase other medications, incurring in additional out-of-pocket expenses [12]. This is further corroborated by a systematic review of the global economic costs of diabetes which reports that a substantial portion of the economic burden of diabetes in low- and middle-income countries was attributed to patients’ out-of-pocket costs, being in general higher for individuals living in household with relatively lower incomes [3].

The costs of management of individuals with diabetes and early stages of diabetic nephropathy (micro and macroalbuminuria) and retinopathy were taken into consideration when estimating the costs of outpatient management overall. Considering our study methods, we would underestimate the costs of more advanced diabetic nephropathy such as ESRD, which is undoubtedly a significant source of costs. Considerable the uncertainty as to the incidence or prevalence of ESRD requiring dialysis among adults with diabetes in Brazil, we believe the prevalence assumed in our study (29%) may still be conservative. Evaluating the national database of dialysis, considering all dialysis procedures performed in Brazil between 2000 and 2012, a study reported 12% prevalence of diabetes as the underlying disease. Although, it has been demonstrated that as much as 42.3% of individuals undergoing dialysis have an underlying diagnosis listed as indeterminate [34]. Furthermore, global estimates considering data from 42 countries report a higher percentage of diabetes as the underlying diagnosis in ESRD patients undergoing dialysis, varying from 11 to 66% [35]. It is worth noting that the use of high-cost medicines for comorbidities associated with ESRD and reported in a previous national study is below the recommended by National guidelines [21]. Finally, the costs of renal transplants were not included, which implies in an underestimation of the real economic burden of diabetic nephropathy.

For procedures related to diabetic retinopathy, we believe our estimates are also conservative. Due to limited data availability on prevalence and healthcare utilization for diabetic retinopathy in Brazil, we considered selected and most common diagnostic and therapeutic procedures. The major limitation is that the parameter used for our estimates was obtained from a panel of experts, with all the caveats inherent to this method, and thus it may not be representative of the country as whole. Indirect costs attributable to impaired vision and blindness were not taken into account. Consequently, these results can also be underestimated.

The costs of hospitalizations are traditionally high, representing the largest component of medical expenditures with diabetes in United Sates (43% of the total medical cost) [36] and European countries (55%) [37]. In this study annual hospitalizations costs due to diabetes, chronic complications and related conditions, accounted for a small fraction of total direct costs (3.84%). This estimate was low even using the national prevalence multiplied by two to include the population with diabetes who are unaware of the disease but might still be hospitalized. Even so, we believe there is a significant underestimation of hospitalizations costs, due to several reasons. First, we only considered hospitalizations occurring in the Public Health System which is used by an estimated 75% of the population, as no database on the private section hospitalizations is available. Second, hospitalization database was developed for administrative-financial functions, particular for reimbursement. It has been well documented in the national literature that hospitalization costs estimated from public reimbursement values in Brazil are significantly lower than costs estimated considering micro-costing methodologies [38]. Additionally, the relative risks obtained from the international literature considered for estimating the etiologic fraction of each medical condition due to diabetes were often considered low. Notwithstanding, this is the first study of diabetes hospitalization costs that took into consideration costs related to hospitalizations due to selected infectious and neoplastic malignancies, for which individuals with diabetes are known to be at increased risk. Even though they represented only 5.3 and 7.4%, respectively, of the total hospitalizations’ costs, while cardiovascular diseases were responsible for 47.9%, these conditions should be considered when attempting to estimate the total economic burden of diabetes-related hospitalizations.

Productivity loss accounted for a significant share of total costs (32.4%). Early retirement was the most important part of lost productivity, reaching a population that would have, on average, 10–15 years of a productive life. These results are in line with recent estimates of the full global economic burden of diabetes in adults aged 20–79 in 2015 which considered data from 184 countries, and reported indirect costs accounting for 34.7% of the total burden [30]. A systematic review of cost-of-illness studies of diabetes revealed that only half of studies estimated indirect costs and the reported proportion of total economic burden varied significantly (12.1% to 57.2%) according to the cost components considered (absenteeism, presenteeism, temporary and permanent disability, early retirement and mortality) and economic and social characteristics of the countries [39]. Our estimates are likely to be conservative as the costs of presenteeism, unemployment from disability, medical licenses and premature deaths were not taken into account. Because we did not include those costs, the society’s perspective cannot be considered complete. Recently, Siqueira et al. (2017) demonstrated that the costs of early death due to cardiovascular diseases in Brazil have compromised 61% of total costs [40], and it would make sense to assume that this would also occur due to the premature deaths caused by diabetes and its complications. Although we understand that these costs are important in a cost-of-illness study, there were problems of incompleteness and inaccuracy in Brazilian mortality data, which led us to decide not to incorporate them.

In addition to the limitations described above, other factors can also be highlighted as contributors to the conservative estimates presented. First, costs of diabetes in the young ages (type 1 diabetes), prediabetes, undiagnosed diabetes, and gestational diabetes mellitus were not considered. Second, we assumed that all individuals with diabetes would be treated in the public health system, but 25% of the Brazilian population has access to the private health care system [6], whose standards of treatment for diabetes and its costs are certainly higher. We assumed that the entire Brazilian population would use the public system since there is evidence suggesting that a significant portion of the individuals with private insurance will still use the public system for certain procedures [41, 42], particularly high-complexity procedures, many of which are not adequately covered by selected private health services. This inability to estimate private system costs is an important limitation. Finally, regarding outpatient management costs, health resource utilization estimates were based on a previous multicenter cost-of-illness study [12]. Despite having included 1000 individuals with diabetes under care at different levels of assistance in 8 medium and large cities and several regions of the country, the sample studied cannot be considered representative of Brazilian adult population with diabetes.

The Brazilian public health system (SUS) provides healthcare to the whole population free of charge, although marked regional inequalities in terms of access and health indicators are still current problems. Medical spending on diabetes will probably be further exacerbated by longer life expectancy and increase in the prevalence, the latter in part attributable to an increase in obesity rates [5] and a reduction in mortality among Brazilians with diabetes [43]. It has been shown in US that diabetes is associated with a substantial reduction in non-disabled years, to a greater extent than the reduction of longevity [44]. A strategic National action plan for confronting with NCDs was established in 2011 in Brazil to promote the development and implementation of effective and evidence-based approaches to the prevention and control of these diseases and their risk factors. One of the initiatives is to carry out surveys with a focus on health interventions and costs of NCDs [45].

To curb the increase in the economic burden of diabetes, cost-effective interventions are needed to efficiently manage the disease and its complications. Unfortunately, limited evidence on cost-effectiveness of primary prevention strategies for diabetes is available, particularly in low- and middle-income countries. This limits the evidence available to decision-makers in many countries in terms of how much resources to invest in primary versus secondary prevention strategies, and selection of the population to be targeted by selected interventions.

This is the first study to estimate the annual economic burden of diabetes in the perspective of the Brazilian society. We demonstrate that the costs of diabetes are significant, making it a major threat to the economic sustainability of the National public and private health systems, as well as a drag on economic growth. Understanding the economic cost of diabetes helps to inform the society and policymakers and will provide important evidence to decision-making on the allocation of scarce health resources, aiming at improved health in Brazil.

## Additional file

**Additional file 1: Table S1.** State level diabetes prevalence and population estimates in adults (20+ years). SUS. Brazil. 2014. **Table S2.** DM and related conditions and relative risks.

## Data Availability

The datasets analyzed in this study are available from the corresponding author (lucianabahia@diabetes.org.br) upon reasonable request.
