# Supplementary material for: Economic burden of diabetes in Brazil in 2014
Source: Diabetol Metab Syndr. 2019 Jul 2;11:54. doi: 10.1186/s13098-019-0448-4 (PMC6604164; doi:10.1186/s13098-019-0448-4)
Supplement: Supplementary file 1 — Additional file 1: Table S1. State level diabetes prevalence and population estimates in adults (20+ years). SUS. Brazil. 2014. Table S2. DM and related conditions and relative risks. [file 13098_2019_448_MOESM1_ESM.docx]

ADDITIONAL TABLES

Table S1. State level diabetes prevalence and population estimates in adults (20+yrs). SUS. Brazil. 2014.

| State | Prevalence (%) | Population with diabetes |
| --- | --- | --- |
|  |  |  |
| Rondônia | 5.0 | 61,000 |
| Acre | 3.3 | 16,038 |
| Amazonas | 4.6 | 115,966 |
| Roraima | 4.0 | 13,200 |
| Pará | 3.8 | 202920 |
| Amapá | 5.0 | 24,250 |
| Tocantins | 5.4 | 55,674 |
| *North region* | *4.3* | *489,048* |
|  |  |  |
| Maranhão | 5.4 | 239,760 |
| Piauí | 5.0 | 111,550 |
| Ceará | 4.9 | 311,052 |
| Rio Grande do Norte | 5.6 | 139,608 |
| Paraíba | 4.5 | 127,125 |
| Pernambuco | 6.2 | 413,788 |
| Alagoas | 6.8 | 154,292 |
| Sergipe | 6.0 | 93,420 |
| Bahia | 5.0 | 539,000 |
| *Notheast region* | *5.4* | *2,129,595* |
|  |  |  |
| Minas Gerais | 6.4 | 992,640 |
| Espírito Santo | 6.1 | 174,765 |
| Rio de Janeiro | 6.4 | 811,328 |
| São Paulo | 7.7 | 2,580,193 |
| *Southeast region* | *7.1* | *4,558,926,504* |
|  |  |  |
| Paraná | 5.7 | 471,447 |
| Santa Catarina | 5.5 | 281,930 |
| Rio Grande do Sul | 7.0 | 602,420 |
| *South region* | *6.2* | *1,355,797* |
|  |  |  |
| Mato Grosso do Sul | 7.8 | 148,512 |
| Mato Grosso | 6.2 | 143,282 |
| Goiás | 6.4 | 306,112 |
| Distrito Federal | 5.8 | 122,032 |
| *Midwest region* | *6.5* | *1,164,030* |
|  |  |  |
| TOTAL* | 6.2 | 9,219,152 |

Note: The prevalence and number of individuals with diabetes were obtained from the National Health Survey - 2013 and the census of the National Institute of Geography and Statistics - 2014 (see methodological section).

* Numbers do not necessarily sum to totals because of rounding

**Table S2.** DM and related conditions and Relative Risks

| **CID Disease** | **Relative Risk** | | | **Reference** |
| --- | --- | --- | --- | --- |
|  | **Men** | **Women** | **Both** |  |
| **Cardiovascular disease** |  |  |  |  |
| I20 Angina pectoris |  |  | 1.89 | (1) |
| I21 Acute myocardial infarction |  |  | 1.74 | (1) |
| I23 Certain current complications following acute myocardial infarction |  |  | 1.74 | (1) |
| I24 Other acute ischaemic heart diseases |  |  | 1.74 | (1) |
| I22 Subsequent myocardial infarction |  |  | 1.74 | (1) |
| I25 Chronic ischaemic heart disease |  |  | 1.89 | (1) |
| I10 Essential (primary) hypertension |  |  | 1.68 | (2) |
| I11 Hypertensive heart disease |  |  | 1.36 | (3) |
| I12 Hypertensive renal disease |  |  | 2.55 | (4) |
| I50 Heart failure |  |  | 1.36 | (3) |
| [I60 Subarachnoid haemorrhage](http://www.medicinanet.com.br/cid10/1763/i60_hemorragia_subaracnoide.htm) |  |  | 1.14 | (1) |
| [I61 Intracerebral haemorrhage](http://www.medicinanet.com.br/cid10/1764/i61_hemorragia_intracerebral.htm) |  |  | 1.14 | (1) |
| I62 Other non-traumatic intracranial haemorrhage |  |  | 1.14 | (1) |
| [I63 Cerebral infarction](http://www.medicinanet.com.br/cid10/1766/i63_infarto_cerebral.htm) |  |  |  | (5) |
| Age group (years) 30-44 |  |  | 5.60 |  |
| 45-59 |  |  | 3.60 |  |
| 60-74 |  |  | 2.10 |  |
| >=75 |  |  | 1.80 |  |
| I65 Occlusion and stenosis of precerebral arteries. not resulting in cerebral infarction |  |  | 1.14 | (1) |
| I66 Occlusion and stenosis of cerebral arteries. not resulting in cerebral infarction |  |  | 1.14 | (1) |
| I67.2 Cerebral atherosclerosis |  |  | 1.76 | (1) |
| [I69 Sequelae of cerebrovascular disease](http://www.medicinanet.com.br/cid10/1772/i69_sequelas_de_doencas_cerebrovasculares.htm) |  |  | 1.76 | (1) |
| G45 Transient cerebral ischemic attacks and related syndromes |  |  |  | (5) |
| Age group (years) 30-44 |  |  | 5.60 |  |
| 45-59 |  |  | 3.60 |  |
| 60-74 |  |  | 2.10 |  |
| 75+ |  |  | 1.80 |  |
|  |  |  |  |  |
| **Renal diseases** |  |  | 2.55 | (4) |
| N04 Nephrotic syndrome |  |  |  |  |
| R77.0 Abnormality of albumin |  |  |  |  |
| R80 Isolated proteinuria |  |  |  |  |
| N17 Acute renal failure |  |  |  |  |
| N18 Chronic kidney disease |  |  |  |  |
| N19 Unspecified kidney failure |  |  |  |  |
|  |  |  |  |  |
| **Eye diseases** |  |  | 3.22 | (4) |
| [H25 Senile cataract](http://www.medicinanet.com.br/cid10/1665/h25_catarata_senil.htm) |  |  |  |  |
| H28 Cataract and other disorders of lens in diseases classified elsewhere |  |  |  |  |
| [H33 Retinal detachments and breaks](http://www.medicinanet.com.br/cid10/1672/h33_descolamentos_e_defeitos_da_retina.htm) |  |  |  |  |
| H34 Retinal vascular occlusions |  |  |  |  |
| [H35.0   Background retinopathy and retinal vascular changes](http://www.medicinanet.com.br/cid10/6057/h350_retinopatias_de_fundo_e_alteracoes_vasculares_da_retina.htm) |  |  |  |  |
| [H35.2](http://www.medicinanet.com.br/cid10/6059/h352_outras_retinopatias_proliferativas.htm)Other proliferative retinopathy |  |  |  |  |
| [H36.0   Retinal disorders in diseases classified elsewhere](http://www.medicinanet.com.br/cid10/6067/h360_retinopatia_diabetica.htm) |  |  |  |  |
| H42 Glaucoma in diseases classified elsewhere |  |  |  |  |
| H54 Visual impairment including blindness (binocular or monocular) |  |  |  |  |
|  |  |  |  |  |
| **Neurological diseases** |  |  |  |  |
| G90 Disorders of autonomic nervous system |  |  | 1.97 | (4) |
| G56 Mononeuropathies of upper limb |  |  | 1.97 | (4) |
| G57 Mononeuropathies of lower limb |  |  | 1.97 | (4) |
| G59.0 Diabetic mononeuropathy |  |  | 1.97 | (4) |
| G63 Polyneuropathy in diseases classified elsewhere |  |  | 1.97 | (4) |
| [G52 Disorders of other cranial nerves](http://www.medicinanet.com.br/cid10/1616/g52_transtornos_de_outros_nervos_cranianos.htm) |  |  | 1.97 | (4) |
| L97 Ulcer of lower limb. not elsewhere classified |  |  | 1.97 | (4) |
| S88 Traumatic amputation of lower leg |  |  |  | (6) |
| Age group (years) 35-44 |  |  | 3.04 |  |
| 45-54 |  |  | 9.82 |  |
| 55-59 |  |  | 22.53 |  |
| 60-64 |  |  | 35.36 |  |
| 65-74 |  |  | 63.52 |  |
| 75+ |  |  | 163.49 |  |
| S98 Traumatic amputation of ankle and foot |  |  |  | (6) |
| Age group (years) 35-44 |  |  | 1.75 |  |
| 45-54 |  |  | 5.01 |  |
| 55-59 |  |  | 7.68 |  |
| 60-64 |  |  | 9.86 |  |
| 65-74 |  |  | 12.15 |  |
| 75+ |  |  | 15.77 |  |
| R02 Gangrene. not elsewhere classified |  |  |  | (6) |
| Age group (years) 35-44 |  |  | 2.07 |  |
| 45-54 |  |  | 6.50 |  |
| 55-59 |  |  | 11.81 |  |
| 60-64 |  |  | 16.49 |  |
| 65-74 |  |  | 23.88 |  |
| 75+ |  |  | 43.92 |  |
| [M86 Osteomyelitis](http://www.medicinanet.com.br/cid10/2069/m86_osteomielite.htm) |  |  | 5.80 | (7) |
| M87 Osteonecrosis |  |  | 5.80 | (7) |
|  |  |  |  |  |
| **Respiratory and urinary infectious diseases** |  |  |  |  |
| N10 Acute tubulo-interstitial nephritis |  |  |  | (8) |
| Age group (years) 18-24 | 1.17 | 1.19 |  |  |
| 25-34 | 1.21 | 1.19 |  |  |
| 35-44 | 1.21 | 1.18 |  |  |
| 45-54 | 1.21 | 1.13 |  |  |
| 55-64 | 1.19 | 1.13 |  |  |
| 65-74 | 1.16 | 1.14 |  |  |
| 75+ | 1.16 | 1.12 |  |  |
| N15.1 Renal and perinephric abscess |  |  |  | (8) |
| Age group (years) 18-24 | 1.17 | 1.19 |  |  |
| 25-34 | 1.21 | 1.19 |  |  |
| 35-44 | 1.21 | 1.18 |  |  |
| 45-54 | 1.21 | 1.13 |  |  |
| 55-64 | 1.19 | 1.13 |  |  |
| 65-74 | 1.16 | 1.14 |  |  |
| 75+ | 1.16 | 1.12 |  |  |
| N30.0 Acute cystitis |  |  |  | (8) |
| Age group (years) 18-24 | 1.17 | 1.19 |  |  |
| 25-34 | 1.21 | 1.19 |  |  |
| 35-44 | 1.21 | 1.18 |  |  |
| 45-54 | 1.21 | 1.13 |  |  |
| 55-64 | 1.19 | 1.13 |  |  |
| 65-74 | 1.16 | 1.14 |  |  |
| 75+ | 1.16 | 1.12 |  |  |
| N30.8 Other cystitis |  |  |  | (8) |
| Age group (years) 18-24 | 1.17 | 1.19 |  |  |
| 25-34 | 1.21 | 1.19 |  |  |
| 35-44 | 1.21 | 1.18 |  |  |
| 45-54 | 1.21 | 1.13 |  |  |
| 55-64 | 1.19 | 1.13 |  |  |
| 65-74 | 1.16 | 1.14 |  |  |
| 75+ | 1.16 | 1.12 |  |  |
| Respiratory infections |  |  | 1.23 | (9) |
| J12 Viral pneumonia. not elsewhere classified |  |  |  |  |
| J13 Pneumonia due to Streptococcus pneumoniae |  |  |  |  |
| J14 Pneumonia due to Haemophilusinfluenzae |  |  |  |  |
| J15 Bacterial pneumonia. not elsewhere classified |  |  |  |  |
| J18 Pneumonia. organism unspecified |  |  |  |  |
|  |  |  |  |  |
| **Neoplasms** |  |  |  |  |
| Breast |  |  | 1.20 | (10) |
| C50 Malignant neoplasm of breast |  |  |  |  |
| D05.9 Carcinoma in situ of breast. unspecified |  |  |  |  |
|  |  |  |  |  |
| Liver and intrahepatic bile ducts |  |  |  |  |
| C22.1 Intrahepatic bile duct carcinoma |  |  | 1.97 | (10) |
| C22.0 Liver cell carcinoma |  |  | 2.31 | (11) |
| C22.7 Other specified carcinomas of liver |  |  | 2.31 | (11) |
| C22.9 Malignant neoplasm of liver and intrahepatic bile ducts - liver. unspecified |  |  | 2.31 | (11) |
|  |  |  |  |  |
| Colorectal |  |  | 1.27 | (10) |
| C18 Malignant neoplasm of colon |  |  |  |  |
| C19 Malignant neoplasm of recto sigmoid junction |  |  |  |  |
|  |  |  |  |  |
| Endometrium |  |  | 1.97 | (10) |
| C54.1 Malignant neoplasm of corpus uteri |  |  |  |  |
| D07.0 Carcinoma in situ of other and unspecified genital organs |  |  |  |  |
|  |  |  |  |  |
| Pancreas |  |  | 1.94 | (12) |
| C25 Malignant neoplasm of pancreas |  |  |  |  |
|  |  |  |  |  |

Notes:

Ref. [2] – RR calculated based on self-report prevalence of hypertension for diabetics in relation to non-diabetics according to the National Health Survey of 2013.

Ref. [3] – RR calculated according Grant (2014) (13).

**References**

1. Sarwar N. Gao P. Seshasai SR. Gobin R. Kaptoge S. Di Angelantonio E. et al. Diabetes mellitus. fasting blood glucose concentration. and risk of vascular disease: a collaborative meta-analysis of 102 prospective studies. Lancet 2010;375(9733):2215-22.

2. IBGE. Pesquisa nacional de saúde - 2013: percepção do estado de saúde. estilos de vida e doenças crônicas: Brasil. grandes regiões e unidades da federação. In; 2014.

3. Kamalesh M. Cleophas TJ. Heart failure due to systolic dysfunction and mortality in diabetes: pooled analysis of 39.505 subjects. J Card Fail 2009;15(4):305-9.

4. Donnan PT. Leese GP. Morris AD. Hospitalizations for people with type 1 and type 2 diabetes compared with the nondiabetic population of Tayside. Scotland: a retrospective cohort study of resource use. Diabetes Care 2000;23(12):1774-9.

5. Jeerakathil T. Johnson JA. Simpson SH. Majumdar SR. Short-term risk for stroke is doubled in persons with newly treated type 2 diabetes compared with persons without diabetes: a population-based cohort study. Stroke 2007;38(6):1739-43.

6. Lombardo FL. Maggini M. De Bellis A. Seghieri G. Anichini R. Lower extremity amputations in persons with and without diabetes in Italy: 2001-2010. PLoS One 2014;9(1):e86405.

7. Al-Mayahi M. Cian A. Kressmann B. de Kalbermatten B. Rohner P. Egloff M. et al. Associations of diabetes mellitus with orthopaedic infections. Infect Dis (Lond) 2016;48(1):70-3.

8. Fu AZ. Iglay K. Qiu Y. Engel S. Shankar R. Brodovicz K. Risk characterization for urinary tract infections in subjects with newly diagnosed type 2 diabetes. J Diabetes Complications 2014;28(6):805-10.

9. Kornum JB. Thomsen RW. Riis A. Lervang HH. Schonheyder HC. Sorensen HT. Diabetes. glycemic control. and risk of hospitalization with pneumonia: a population-based case-control study. Diabetes Care 2008;31(8):1541-5.

10. Tsilidis KK. Kasimis JC. Lopez DS. Ntzani EE. Ioannidis JP. Type 2 diabetes and cancer: umbrella review of meta-analyses of observational studies. Bmj 2015;350:g7607.

11. Wang C. Wang X. Gong G. Ben Q. Qiu W. Chen Y. et al. Increased risk of hepatocellular carcinoma in patients with diabetes mellitus: a systematic review and meta-analysis of cohort studies. Int J Cancer 2012;130(7):1639-48.

12. Ben Q. Xu M. Ning X. Liu J. Hong S. Huang W. et al. Diabetes mellitus and risk of pancreatic cancer: A meta-analysis of cohort studies. Eur J Cancer 2011;47(13):1928-37.

13. Grant RL. Converting an odds ratio to a range of plausible relative risks for better communication of research findings. BMJ 2014;348(f7450).
